# Health-related quality of life of moderate and severe haemophilia patients: Results of the haemophilia-specific quality of life index in Korea

**DOI:** 10.1371/journal.pone.0238686

**Published:** 2020-09-03

**Authors:** Hee Jo Baek, Young Shil Park, Ki Young Yoo, Jin-Hye Cha, Young-Joo Kim, Kun Soo Lee

**Affiliations:** 1 Department of Pediatrics, Chonnam National University Hwasun Hospital, Chonnam National University Medical School, Gwangju, South Korea; 2 Department of Pediatrics, Kyung Hee University Hospital at Gangdong, Seoul, South Korea; 3 Korea Hemophilia Foundation, Seoul, South Korea; 4 Pfizer Pharmaceuticals Korea Ltd., Seoul, South Korea; 5 Department of Pediatrics, Kyungpook National University Medical School, Kyungpook National University Hospital, Daegu, South Korea; Erasmus MC Desiderius School, NETHERLANDS

## Abstract

The assessment of health-related quality of life (HRQoL) as a patient-reported outcome provides information about the patients’ general well-being as well as the effects of the disease and its treatment. This study aimed to investigate HRQoL using both generic and haemophilia-specific QoL instruments and to assess the clinical factors associated with HRQoL among haemophilia patients in Korea. In this cross-sectional, multicenter, observational study, moderate-to-severe haemophilia patients aged 8–64 years were recruited between November 2012 and September 2013. The EQ-5D Questionnaire, EQ Visual Analogue Scale, and Haemophilia-Specific QoL (Haemo-QoL) Questionnaire (Haemo-QoL for 8–16 years and Haemo-A-QoL for ≥17 years) were used to assess HRQoL. A total of 605 participants with a mean age of 29.32 ± 12.62 years were enrolled. The mean Haemo-QoL scores revealed significant differences by age group (children vs. adolescent vs. adult, 26.44 ± 11.3 vs. 28.88 ± 11.1 vs. 38.43 ± 17.7, respectively, *p* < 0.001). “Sports and leisure,” “family planning,” and “view” in adults and “perceived support,” “friends,” and “dealing” in children and adolescents were identified as the domains with the greatest HRQoL impairments. HRQoL was significantly impaired in patients with the following clinical factors: hepatitis, haemophilia-induced disability, bleeding experiences within the last 6 months, joint bleedings within the last 6 months, and haemophilic arthropathy. According to the multivariate regression analysis, HRQoL showed a negative association with the presence of haemophilia-induced disability (β = 0.222, *p* < 0.0001), bleeding experiences within the last 6 months (β = 0.098, *p* = 0.010), and haemophilic arthropathy (β = 0.212, *p* < 0.0001). HRQoL decreased in patients with older age and impaired clinical conditions among moderate-to-severe haemophilia patients in Korea. These study findings may provide significant insights into the adequate haemophilia management using patient-reported measurements.

## Introduction

Haemophilia A and B are inherited bleeding disorders due to a deficiency of coagulation factors. Bleeding episodes are the main consequence of haemophilia. Chronic arthropathy caused by repeated joint bleeding can lead to joint pain, reduction in joint range of motion, and permanent joint damage and disability [1–3]. Moreover, internal bleeding in some parts of the body, such as the brain, throat, and abdomen, can be life-threatening and cause disability in the absence of prompt treatment [3,4].

Clinical conditions and disease progression of haemophilia can be evaluated by performing joint examination and/or monitoring bleeding frequency and severity [1]. However, these objective clinical measurements limit the functional status assessment of haemophilia patients.

The assessment of health-related quality of life (HRQoL) in haemophilia as a patient-reported outcome provides direct information about haemophilia patients’ general well-being as well as the effects of haemophilia and treatment outcomes [1]. The generic assessment of HRQoL in haemophilia started in 1990 [5–8]. Subsequently, both generic and disease-specific instruments have been used to assess the HRQoL of haemophilia patients. The EQ-5D Questionnaire and the Medical Outcomes Study Short Form-36 are generic HRQoL instruments [9,10].

As not much research has been conducted on this topic in the past, not much is known about the HRQoL of Korean haemophilia patients. Therefore, this study aimed to investigate HRQoL using both generic and haemophilia-specific QoL instruments and to assess clinical factors associated with HRQoL among moderate-to-severe moderate to severe haemophilia A and B patients in Korea. This study summarizes the first application of the validated Korean version of Haemo-QoL and Haemo-A-QoL, which are haemophilia-specific QoL instruments for children and adults, respectively.

## Materials and methods

This study was approved by the Institutional Review Board (IRB) of each participating center before study conduction, and all participating patients read and signed the written consent forms prior to study participation. The IRB were the Kyoungpook National University Hospital IRB, Kyung Hee University Hospital at Gangdong IRB, and Chonnam National University Hwasun Hospital IRB. For the ethics approval of the two haemophilia foundations, the study approval was received from the Kyoungpook National University Hospital IRB since no internal IRB was established at haemophilia foundations.

### Study design

This cross-sectional, noninterventional, observational study was conducted at three nationwide hospitals and two haemophilia foundations located in South Korea between November 2012 and September 2013. Both medical chart review and patient survey were utilized for data collection.

### Eligibility and exclusion criteria

The eligibility criteria were as follows: (i) male with moderate-to-severe haemophilia A or B (haemophilia severity was categorized as moderate if the factor activity was 1%–5% and severe if it was <1% of the normal activity); (ii) 8–64 years old; and (iii) receiving at least 90% of haemophilic care at the participating centers. The exclusion criteria were as follows: (i) presence of any bleeding disorders other than haemophilia A or B; (ii) a positive human immunodeficiency virus (HIV) test result; or (iii) presence of any other severe, acute/chronic, or mental illness.

### Data collection

Potentially eligible patients were identified, and all eligible patients provided informed consent for inclusion in the study. Demographic and clinical characteristics and laboratory data were collected by the physicians from the patients’ medical records. Data on haemophilic arthropathy in target joints of any side of the knees, ankles, elbows, wrists, or shoulders was collected as recorded in medical chart. Haemophilia-induced disabilities included physical disability, intellectual disability, and brain lesions, as confirmed by the physicians. Comorbidities included myocardial infarction, congestive heart disease, diabetes, chronic pulmonary disease, peptic ulcer, renal disease, liver disease, connective tissue disease, and cancer. Regarding the treatment method types, on-demand treatment was defined to infuse factor concentrates during bleeding. Maintenance treatment was defined as the regular infusion of factor concentrates at least one time per week during the last 6 months from the time of enrollment in the study.

### Instruments

All patients completed the self-reported questionnaires to measure HRQoL. The validated Korean version of EQ-5D-3L, a generic HRQoL instrument, was applied to all patients [11]. Further, the validated Korean versions of the following haemophilia-specific QoL assessment instruments were used: Haemo-QoL and Haemophilia QoL Questionnaire for Adults (Haemo-A-QoL) (Haemo-QoL Long Form Kids II for the children group [8–12 years], Haemo-QoL Long Form Kids III for the adolescent group [13–16 years], and Haemo-A-QoL for the adult group [≥17 years]) [10–12].

#### Generic QoL instruments

EQ-5D-3L, which has been widely used for utility-based HRQoL measurements, was used in this study. EQ-5D-3L consists of the following five domains: mobility, self-care, anxiety/depression, usual activities, and pain/discomfort, with three levels of responses (no problems, some problems, and extreme problems). Many countries developed country-specific value sets for EQ-5D-3L. Here, we used the Korean value set developed by the Korea Institute for Health and Social Affairs for the score conversion to the index value [13]. The following equation was used to obtain EQ-5D-3L summary index values [13]: Final EQ-5D-3L index value = 1-(0.164+0.003×M2+0.274×M3+0.058×SC2+0.078×SC3+0.045×UA2+0.134×UA3+0.049×PD2+0.132×PD3+0.044×AD2+0.102×AD3+0.345×N3+0.014×I2sq). The EQ-5D-3L index value ranges from −0.229 (possible score of the worst imaginable health status) to 1 (possible score of the best imaginable health status).

The EQ VAS addresses self-rated health on a vertical, VAS, with scores ranging from 0 (the worst imaginable health status) to 100 (the best imaginable health status). High scores on both generic QoL instruments correspond to enhanced HRQoL.

#### Haemophilia-specific QoL instruments

For children aged 8–12 years, Haemo-QoL Long Form Kids II, consisting of 10 dimensions (physical health, feeling, view of yourself, family, friends, support, others, sport, dealing, and treatment) with a total of 64 questions, was applied, and for adolescents aged 13–16 years, Haemo-QoL Long Form Kids III containing 12 dimensions (an additional 2 dimensions, relationships and future, were added to the children’s version) with 77 questions was applied. Haemo-A-QoL, which is designed for adults aged 17 years or older, consists of 46 items pertaining to 10 dimensions (physical health, feeling, view, sport, work, dealing, treatment, future, family planning, and relationship). Each dimension indicates the following meanings: ‘physical health’ dimension is about the children’s pain, bleeding, and restriction of movement, etc., due to haemophilia; ‘feeling’ dimension evaluates the children’s feelings on their haemophilia; ‘view of yourself’ dimension means how the children perceive themselves; ‘family’ and ‘friends’ dimensions are regarding the interaction with family and friends, respectively; ‘support’ is about how children perceive the support they received; ‘others’ dimension indicates the interaction with others; ‘sports’ dimension is about the restrictions from sports; ‘dealing’ dimension means how children deal with their haemophilia; ‘treatment’ dimension is about their factor injection etc.; ‘future’ and ‘relationship’ dimensions are about their feeling about the future and having a girlfriend (intersexual friend), respectively.

Across all age groups, haemophilia-specific questions fall under five levels, and the sum of the scores for all dimensions in each age group can be combined to produce a total Haemo-QoL score for children and adolescents and a total Haemo-A-QoL score for adults. Raw scores were converted to a scale from 0 to 100, and high scores represent low HRQoL [12].

### Statistical analysis

Descriptive data analysis was performed to analyze the demographic/clinical variables and HRQoL in haemophilia patients. Of the data collected, continuous data were presented as basic statistics (number of observations, averages, and standard deviations) and categorical variables were presented as frequency and percentage (%). HRQoL was compared according to the demographic and clinical variables. The Student’s *t*-test and Mann–Whitney *U* test were used to examine differences according to the normality of distribution for the variable. For multiple comparisons, the Bonferroni correction was utilized. To assess whether the generic and disease-specific instruments agree, the Pearson’s correlation coefficients were calculated. Multiple linear regression analyses were performed to determine the clinical factors associated with HRQoL using backward elimination. All final significance levels reported were two-tailed, and statistical significance was estimated at *p* ≤0.05. The Statistical Package for Social Sciences program (version 20.0) was used for statistical analysis.

## Results

### Demographic and clinical characteristics

Table 1 shows the demographic and clinical characteristics of the participants. Of the 605 participants included in this study, 506 and 99 had haemophilia A and B, respectively. The mean (± standard deviation, SD) age of the participants was 29.32 (± 12.62) years (range, 8–64 years). Twenty-three participants with persistent inhibitors of more than 1 BU/mL were included. More than three-fourths of the participants had haemophilic arthropathy, and the most commonly affected joint was the ankle. Of the participants enrolled, 88.6% had severe haemophilia and 90% had more than one bleeding episode. Of those with bleeding episodes, 91.4% had experienced joint bleedings within the last 6 months. Haemophilia-induced disability was observed in 34.2% of all participants.

**Table 1:** Demographic and clinical characteristics of the participants.

	Number (%)
**Age at enrollment in years, mean ± SD**	29.32 ± 12.62
8–12	47 (7.8)
13–16	53 (8.8)
≥17	505 (83.5)
**Type of factor deficiency**	
VIII (haemophilia A)	506 (83.6)
IX (haemophilia B)	99 (16.4)
**Haemophilia severity on diagnosis**	
Severe	536 (88.6)
Moderate	69 (11.4)
**History of FVIII or FIX inhibitors titer ≥1 BU/mL**	103 (17.0)
Persistent FVIII or FIX inhibitors	23 (3.8)
**Hepatitis**	
Hepatitis C	140 (23.1)
Hepatitis B	13 (2.1)
Both B and C	13 (2.1)
**Bleeding experiences within the last 6 months**^a^	544 (90.0)
Joint	497 (91.4)
Muscle	218 (36.0)
Face	108 (17.9)
Hematuria	22 (3.6)
**Severe bleeding within the last 1 year**^a^	27 (4.5)
Central nervous system (CNS)	3 (0.5)
Gastrointestinal tract	20 (3.3)
Neck/throat	2 (0.3)
Severe bleeding with serious injury or trauma	5 (0.8)
**Haemophilic arthropathy**^a^	469 (77.5)
Ankle joint	304 (64.8)
Elbow joint	227 (48.4)
Knee joint	223 (47.5)
Shoulder joint	49 (10.4)
Wrist joint	8 (1.7)
Other joints	43 (9.2)
**Haemophilia-induced disability**^a^	207 (34.2)
Physical disability	207 (100.0)
Intellectual disability	1 (0.5)
CNS lesions	1 (0.5)
**Comorbidity**	128 (21.2)
**Type of treatment methods**	
On-demand	298 (49.3)
Maintenance	307 (50.7)
Age at the first administration of coagulation factor concentrates	9.25 ± 10.90
Number of target joints^a^, mean ± SD	3.45 ± 1.99

^a^Multiple answers were possible

### HRQoL

The mean (± SD) total Haemo-QoL and Haemo-A-QoL scores, EQ-5D-3L index value, and EQ VAS score of all patients were 36.66 (± 17.29), 0.69 (± 0.15), and 70.22 (± 19.75), respectively.

**Fig 1:**
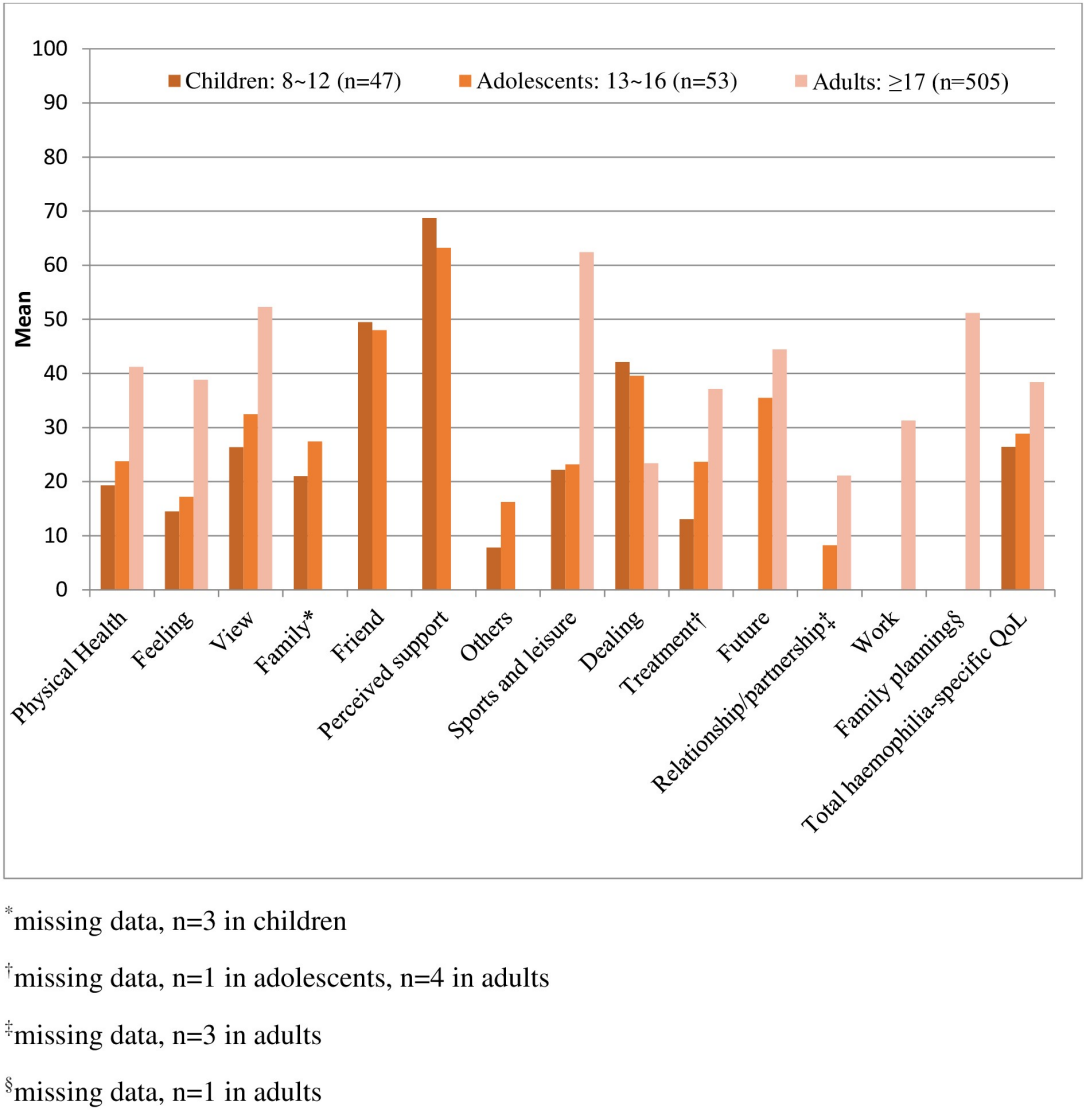
The mean Haemo-QoL questionnaire and Haemo-A-QoL questionnaire scores by age group.

Fig 1 shows the mean Haemo-QoL and Haemo-A-QoL questionnaire scores in total and by dimension according to age group. In the adult group, the dimension with the highest mean score, which indicates poor HRQoL, was “sports and leisure” (mean ± SD: 62.43 ± 26.9), followed by “view” (mean ± SD, 52.30 ± 22.9), and “family planning” (mean ± SD, 51.19 ± 46.8). “Relationships and partners” was the least impaired dimension among adults (mean ± SD, 21.15 ± 26.5). In the children and adolescents group, higher scores for the “perceived support” dimension indicated that they perceived insufficient support from others (mean ± SD in the children group, 68.75 ± 25.6; mean ± SD in the adolescent group, 63.21 ± 26.8). Furthermore, interaction with their “friends” was hindered in these groups (mean ± SD in the children group, 49.47 ± 25.1; mean ± SD in the adolescent group, 48.00 ± 19.3).

HRQoL was assessed according to the patients’ baseline characteristics. For all measurements, HRQoL significantly differed by age group (*p* < 0.01). When applying the Bonferroni correction in comparing age groups, patients aged 17 years or older reported more impaired HRQoL than the younger age groups; lower EQ-5D-3L and EQ VAS values were observed in patients aged 17 years or older compared with patients aged 8–12 years (*P* = 0.007 *and P<*0.001, respectively) and higher scores of Haemo-A-QoL in patients aged 17 years or more were observed compared to Haemo-QoL scores in patients aged 8–12 years and 13–16 years (*P* < 0.001).

The Haemo-QoL and Haemo-A-QoL scores showed good discrimination between patients with hepatitis infection and others (mean ± SD, 40.29 ± 18.52 vs. 35.29 ± 16.62, respectively, *p* = 0.001), between patients with persistent inhibitors and others (mean ± SD, 45.69 ± 17.24 vs. 36.31 ± 17.21, respectively, *p* = 0.011), and between patients with severe haemophilia and those with moderate haemophilia (mean ± SD, 37.47 ± 17.06 vs. 30.37 ± 17.84, respectively, *p* = 0.001). Additionally, impaired HRQoL, per Haemo-QoL and Haemo-A-QoL, was observed in patients with haemophilia-induced disabilities (mean ± SD, 44.04 ± 18.28 vs. 32.83 ± 15.43, *p* < 0.001), comorbidities (mean ± SD, 40.42 ± 18.72 vs. 35.66 ± 16.76, *p* = 0.006), or haemophilic arthropathy (mean ± SD, 39.58 ± 17.19 vs. 26.60 ± 13.45, *p* < 0.001) as opposed to those without these conditions. Patients with bleeding episodes in the last 6 months (mean ± SD, 37.60 ± 17.41 vs. 28.36 ± 13.70, *p* < 0.001) or joint bleeding in the last 6 months (mean ± SD, 38.36 ± 17.33 vs. 28.84 ± 14.81, *p* < 0.001) showed more impairment in HRQoL, as measured by Haemo-QoL and Haemo-A-QoL, than those without bleeding episodes.

Similarly, HRQoL as assessed by EQ-5D-3L was more significantly impaired in patients with the following clinical factors compared with those without: hepatitis, haemophilia-induced disability, bleeding experiences within the last 6 months, joint bleedings within the last 6 months, and haemophilic arthropathy (Table 2).

**Table 2:** Comparison of health-related quality of life according to patients’ characteristics.

	EQ-5D-3L	*P-value*	EQ VAS	*P-value*	Haemo-QoL and Haemo-A-QoL	*P-value*
Age, years						
8–12^a^	0.74±0.11^c^	*0*.*007*	80.26±18.22^c^	*<0*.*001*	26.44±11.32^c^	*<0*.*001*
13–16^b^	0.71±0.14		74.20±20.11		28.88±11.06^c^	
≥17^c^	0.68±0.16^a^		68.91±19.57^a^		38.43±17.74^a,b^	
Severity of haemophilia						
Severe	0.68±0.15	*0*.*552*	70.22±19.42	*0*.*986*	37.47±17.06	*0*.*001*
Moderate	0.70±0.17		70.18±22.28		30.37±17.84	
Hepatitis						
Yes	0.65±0.17	*0*.*001*	67.40±20.07	*0*.*032*	40.29±18.52	*0*.*001*
No	0.70±0.17		71.31±19.54		35.29±16.62	
History of FVIII or FIX inhibitors titer ≥1 BU/mL						
Yes	0.68±0.15	*0*.*788*	69.28±20.04	*0*.*600*	39.00±18.03	*0*.*132*
No	0.69±0.15		70.41±19.71		36.18±17.11	
Persistent FVIII or FIX inhibitors titer ≥1 BU/mL						
Yes	0.63±0.16	*0*.*117*	64.14±20.89	*0*.*151*	45.69±17.24	*0*.*011*
No	0.69±0.15		70.44±19.69		36.31±17.21	
Haemophilia-induced disability						
Yes	0.65±0.16	*<0*.*001*	66.08±20.29	*<0*.*001*	44.04±18.28	*<0*.*001*
No	0.70±0.15		72.45±19.12		32.83±15.43	
Comorbidities						
Yes	0.66±0.15	*0*.*042*	68.43±20.62	*0*.*250*	40.42±18.72	*0*.*006*
No	0.69±0.15		70.71±19.50		35.66±16.76	
Bleeding experiences within the last 6 months						
Yes	0.68±0.16	*0*.*001*	69.48±19.96	*0*.*008*	37.60±17.41	*<0*.*001*
No	0.73±0.13		76.65±16.65		28.36±13.70	
Joint bleeding within the last 6 months						
Yes	0.68±0.16	*0*.*001*	69.03±19.96	*0*.*002*	38.36±17.33	*<0*.*001*
No	0.73±0.13		75.70±17.85		28.84±14.81	
Haemophilic arthropathy						
Yes	0.67±0.16	*<0*.*001*	68.44±19.50	*<0*.*001*	39.58±17.19	*<0*.*001*
No	0.73±0.12		76.39±19.44		26.60±13.45	
Type of treatment methods						
On-demand	0.68±0.16	*0*.*361*	68.19±21.28	*0*.*015*	36.70±17.90	*0*.*958*
Maintenance	0.69±0.15		72.19±17.97		36.63±16.70	

Superscripts indicate significant univariate differences between groups by the Bonferroni adjusted multiple comparisons

Significant correlations were verified between the generic and disease specific instruments. As there is an opposite direction between the value of EQ-5D-3L index/EQ VAS and Haemo-QoL, inverse correlations were found (Table 3).

**Table 3:** Pearson correlations amongst measurements.

	EQ-5D-3L index value	EQ VAS	Haemo-QoL and Haemo-A-QoL total score
EQ-5D-3L index value	1		
EQ VAS	0.324***	1	
Haemo-QoL and Haemo-A-QoL total score	-0.373***	-0.491***	1

***p < .001

According to the multiple linear regression analysis, HRQoL measured using both EQ-5D-3L and Haemo-QoL or Haemo-A-QoL showed negative association with bleeding experiences within the last 6 months, the presence of haemophilia-induced disability and haemophilic arthropathy (Table 4).

**Table 4:** Multivariate analyses for clinical factors associated with health-related quality of life according to the instrument used.

Variables	B	SE^a^	β^b^	*P-value*
**EQ-5D-3L**				
** Hepatitis**				
** Yes (no)**	-0.026	0.015	-0.076	*0*.*081*
** Haemophilia-induced disability**				
** Yes (no)**	-0.035	0.014	-0.110	*0*.*014*
** Haemophilic arthropathy**				
** Yes (no)**	-0.043	0.016	-0.117	*0*.*008*
**EQ VAS**				
** Age**				
** 8–12 (≥ 17)**	7.050	3.214	2.194	*0*.*029*
** Haemophilia-induced disability**				
** Yes (no)**	-4.116	1.792	-2.297	*0*.*022*
** Bleeding experiences within the last 6 months**				
** Yes (no)**	-5.434	2.686	-2.023	*0*.*044*
** Haemophilic arthropathy**				
** Yes (no)**	-3.806	2.207	-1.725	*0*.*085*
**Haemo-QoL and Haemo-A-QoL**				
** Persistent FVIII or FIX inhibitors titer ≥1 BU/mL**				
** Yes (no)**	5.883	3.402	0.065	*0*.*084*
** Haemophilia-induced disability**				
** Yes (no)**	8.080	1.456	0.222	*<0*.*001*
** Bleeding experiences within the last 6 months**				
** Yes (no)**	5.646	2.195	0.098	*0*.*010*
** Haemophilic arthropathy**				
** Yes(no)**	8.753	1.685	0.212	*<0*.*001*

^a^SE, standard error

B, coefficient

^b^*β*, standardized coefficient

## Discussion

A large number of haemophilia A and B patients participated in this study, thus allowing us to summarize the overall HRQoL status. This study revealed that HRQoL decreased in patients with older age and impaired clinical conditions among moderate-to-severe haemophilia patients in Korea.

The mean EQ-5D-3L index value and EQ VAS in all haemophilia patients in this study were presented as 0.68 ± 0.15 and 70.22 ± 19.75, respectively, which were similar to the results from two previous studies reporting EQ-5D-3L index values, ranging from 0.66 to 0.69, and concluding that the QoL of haemophilia patients was significantly lower than that of the general population [9,14].

Compared with the findings on EQ-5D-3L index of chronic diseases from the 2005 Korea National Health and Nutrition Examination Survey [15], the index value of haemophilia patients in our study was relatively higher than that of patients with stroke (0.51–0.58) and was similar to that of patients with renal failure (0.66–0.77), but it was lower than that of patients with angina pectoris (0.73–0.84) and arthritis (0.76–0.86).

In the study on the development and testing of the Haemo-QoL, the instrument showed acceptable internal consistency, retest reliability, and sufficient discriminant and convergent validity [12]. Haemo-A-QoL showed satisfactory psychometric characteristics (reliability, validity) in a validation study [16]. There are several studies in which Haemo-QoL and Haemo-A-QoL have been utilized as haemophilia-specific QoL instruments [2, 17–20]. A multinational study of children aged 4–16 years with severe haemophilia was conducted in Western European countries (Germany, Italy, France, Spain, the Netherlands, and the UK). Older children aged 8–16 years had higher impairments in the so-called “social” dimensions, such as “perceived support” and “friends” [2]. These results were very similar to the results of this study. In this study, results of the children and adolescent groups indicated that they received insufficient support from others in the “perceived support” dimension (children group, 68.75 ± 25.6; adolescent group, 63.21 ± 26.8) and interactions with their “friends” were impaired (children group, 49.47 ± 25.1; adolescent group, 48.00 ± 19.3). In some psychometric studies of adult haemophilia [14,17,19], “physical health” and “sports and leisure” Haemo-A-QoL domains were found to have the highest degree of HRQoL impairment. In the adult group in our study, the “sports and leisure” dimension had the greatest HRQoL impairment (mean score, 62.43 ± 26.9), followed by “family planning,” “view,” “future,” and “physical health.” Moreover, in a study conducted in a single center in Turkey [20], the worst Haemo-A-QoL dimension in adults (>16 years) was “sports,” consistent with the results of this study.

Previous studies have reported that older age, frequent acute bleeding, patients with severe haemophilia and Pettersson score >21, presence of inhibitors to clotting factors, moderate-to-severe joint manifestations, hepatitis C diagnosis, presence of arthropathy, and positive HIV test result were associated with reduced HRQoL among haemophilia patients [9,14,21–26]. With respect to the associated factors, our study strengthened the relationship between several factors and HRQoL in patients with haemophilia. Univariate analysis of the study population revealed that older age, presence of hepatitis, haemophilia-induced disability, bleeding experience and joint bleeding in the last 6 months, and haemophilic arthropathy were associated with impaired HRQoL, using both generic and haemophilia-specific measurements. Clinical factors such as severe disease, presence of persistent inhibitors, presence of comorbidity, and on-demand treatment were associated with reduced HRQoL, as assessed by generic or haemophilia-specific measurements.

Multivariate analysis revealed that consequent physical conditions affected by haemophilia, such as haemophilia-induced disabilities, bleeding experiences, and haemophilic arthropathy, were significantly associated with impaired HRQoL, when measured using both EQ-5D-3L and haemophilia-specific measurements.

In a recent multinational phase 3 clinical trial, which included adults with severe haemophilia A or B, Haemo-A QoL demonstrated higher convergent validity than EQ-5D-3L, including the subdimension scores and total score [16]. In this study, EQ-5D-3L index/EQ VAS and Haemo-QoL, significant correlations were found. Furthermore, clinical conditions associated with impaired HRQoL were similar to those associated with impaired EQ-5D-3L and/or EQ VAS.

This study has some limitations. First, relatively compliant patients may have been selectively included in this study because filling out self-reported questionnaires required some time. Second, the causal association between the clinical factors and HRQoL may have been overemphasized or underemphasized owing to the cross-sectional nature of the study.

However, this study is noteworthy since it is the first nationwide multicenter study that investigates HRQoL among haemophilia patients in Korea using a structured, validated measurement tool. This study reported impairments on specific HRQoL dimensions measured using a haemophilia-related QoL instrument that differed according to age group and presented the associations between clinical factors and HRQoL in Korean haemophilia patients. This perspective may provide significant understanding for the adequate management of moderate-to-severe haemophilia patients.

## Conclusions

To the best of our knowledge, this study was the first study to assess the QoL in moderate-to-severe haemophilia patients in Korea. This study revealed the current status on not only generic, but also disease-specific QoL. Moreover, it demonstrated the clinical factors associated with the QoL that could provide a strong foundation for the development of treatment strategies for more effective haemophilia management in line with the patients’ perspective.
